# Examination of the performance of machine learning-based automated coronary plaque characterization by near-infrared spectroscopy–intravascular ultrasound and optical coherence tomography with histology

**DOI:** 10.1093/ehjdh/ztaf009

**Published:** 2025-03-04

**Authors:** Retesh Bajaj, Ramya Parasa, Alexander Broersen, Thomas Johnson, Mohil Garg, Francesco Prati, Murat Çap, Nathan Angelo Lecaros Yap, Medeni Karaduman, Carol Ann Glorioso Rexen Busk, Stephanie Grainger, Steven White, Anthony Mathur, Hector M García-García, Jouke Dijkstra, Ryo Torii, Andreas Baumbach, Helle Precht, Christos V Bourantas

**Affiliations:** Department of Cardiology, Barts Heart Centre, Barts Health NHS Trust, West Smithfield, London EC1A 7BE, UK; Centre for Cardiovascular Medicine and Device Innovation, William Harvey Research Institute, John Vane Science Centre, Charterhouse Square, London EC1M 6BQ, UK; Department of Cardiology, Ottawa Heart Institute, Ottawa, ON, Canada; Department of Cardiology, Barts Heart Centre, Barts Health NHS Trust, West Smithfield, London EC1A 7BE, UK; Centre for Cardiovascular Medicine and Device Innovation, William Harvey Research Institute, John Vane Science Centre, Charterhouse Square, London EC1M 6BQ, UK; The Essex Cardiothoracic Centre, Basildon, UK; Division of Image Processing, Department of Radiology, Leiden University Medical Center, Leiden, The Netherlands; Department of Cardiology, University Hospitals Bristol and Weston NHS Foundation Trust, Bristol, UK; Department of Cardiology, Medstar Cardiovascular Research Network, Medstar Washington Hospital Center, Washington, DC, USA; Cardiovascular Sciences Department, Interventional Cardiology Unit, San Giovanni Addolorata Hospital, Rome, Italy; Centro per la Lotta Contro L’Infarto—CLI Foundation, Rome, Italy; Department of Cardiology, Barts Heart Centre, Barts Health NHS Trust, West Smithfield, London EC1A 7BE, UK; Centre for Cardiovascular Medicine and Device Innovation, William Harvey Research Institute, John Vane Science Centre, Charterhouse Square, London EC1M 6BQ, UK; Department of Cardiology, Faculty of Medicine, Yuzuncu Yil University, Van, Turkey; Health Sciences Research Centre, UCL University College, Niels Bohrs Allé 1, 5230 Odense M, Denmark; Department of Radiology, Lillebaelt Hospital, University Hospitals of Southern Denmark, Sygehusvej 24, 6000 Kolding, Denmark; Department of Regional Health Research, University of Southern Denmark, Campusvej 55, DK-5230 Odense M Denmark; Infraredx, Bedford, MA, USA; Biosciences Institute, Newcastle University, Newcastle, UK; Department of Cardiology, Barts Heart Centre, Barts Health NHS Trust, West Smithfield, London EC1A 7BE, UK; Centre for Cardiovascular Medicine and Device Innovation, William Harvey Research Institute, John Vane Science Centre, Charterhouse Square, London EC1M 6BQ, UK; Department of Cardiology, Medstar Cardiovascular Research Network, Medstar Washington Hospital Center, Washington, DC, USA; Department of Radiology, Leiden University Medical Center, Leiden, The Netherlands; Department of Mechanical Engineering, University College London, London, UK; Department of Cardiology, Barts Heart Centre, Barts Health NHS Trust, West Smithfield, London EC1A 7BE, UK; Centre for Cardiovascular Medicine and Device Innovation, William Harvey Research Institute, John Vane Science Centre, Charterhouse Square, London EC1M 6BQ, UK; Health Sciences Research Centre, UCL University College, Niels Bohrs Allé 1, 5230 Odense M, Denmark; Department of Radiology, Lillebaelt Hospital, University Hospitals of Southern Denmark, Sygehusvej 24, 6000 Kolding, Denmark; Department of Regional Health Research, University of Southern Denmark, Campusvej 55, DK-5230 Odense M Denmark; Department of Cardiology, Barts Heart Centre, Barts Health NHS Trust, West Smithfield, London EC1A 7BE, UK; Centre for Cardiovascular Medicine and Device Innovation, William Harvey Research Institute, John Vane Science Centre, Charterhouse Square, London EC1M 6BQ, UK

**Keywords:** Near-infrared spectroscopy–intravascular ultrasound, Optical coherence tomography, Plaque composition, Machine learning, Histology

## Abstract

**Aims:**

Near-infrared spectroscopy–intravascular ultrasound (NIRS–IVUS) and optical coherence tomography (OCT) can assess coronary plaque pathology but are limited by time-consuming and expertise-driven image analysis. Recently introduced machine learning (ML)-classifiers have expedited image processing, but their performance in assessing plaque pathology against histological standards remains unclear. The aim of this study is to assess the performance of NIRS–IVUS–ML-based and OCT–ML-based plaque characterization against a histological standard.

**Methods and results:**

Matched histological and NIRS–IVUS/OCT frames from human cadaveric hearts were manually annotated and fibrotic (FT), calcific (Ca), and necrotic core (NC) regions of interest (ROIs) were identified. Near-infrared spectroscopy–intravascular ultrasound and OCT frames were processed by their respective ML classifiers to segment and characterize plaque components. The histologically defined ROIs were overlaid onto corresponding NIRS–IVUS/OCT frames and the ML classifier estimations were compared with histology. In total, 131 pairs of NIRS–IVUS/histology and 184 pairs of OCT/histology were included. The agreement of NIRS–IVUS–ML with histology [concordance correlation coefficient (CCC) 0.81 and 0.88] was superior to OCT–ML (CCC 0.64 and 0.73) for the plaque area and burden. Plaque compositional analysis showed a substantial agreement of the NIRS–IVUS–ML with histology for FT, Ca, and NC ROIs (CCC: 0.73, 0.75, and 0.66, respectively) while the agreement of the OCT–ML with histology was 0.42, 0.62, and 0.13, respectively. The overall accuracy of NIRS–IVUS–ML and OCT–ML for characterizing atheroma types was 83% and 72%, respectively.

**Conclusion:**

NIRS–IVUS–ML plaque compositional analysis has a good performance in assessing plaque components while OCT–ML performs well for the FT, moderately for the Ca, and has weak performance in detecting NC tissue. This may be attributable to the limitations of standalone intravascular imaging and to the fact that the OCT–ML classifier was trained on human experts rather than histological standards.

## Graphical Abstract

**Graphical Abstract:**
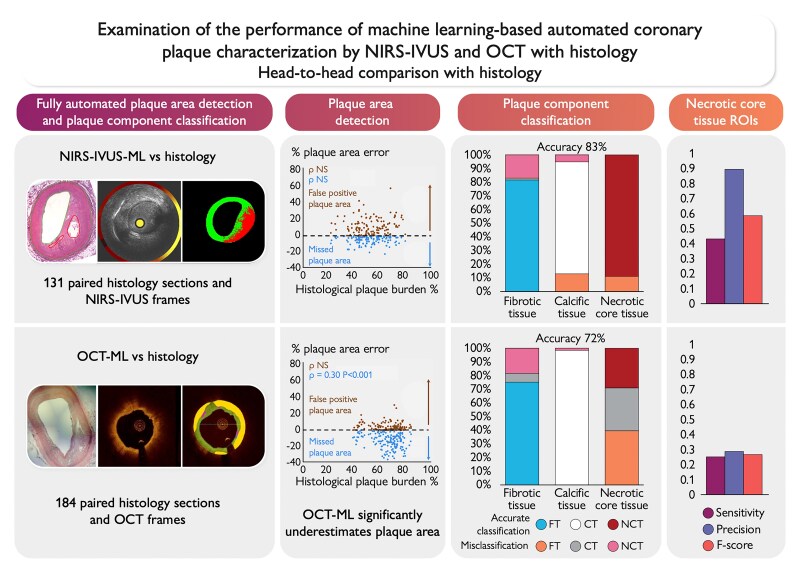
A head-to-head comparison between NIRS–IVUS and OCT-based machine learning classifiers and histology for fully automated plaque component classification.

## Introduction

Coronary intravascular imaging enables a detailed assessment of the atherosclerotic plaque pathology that is essential in treatment planning and vulnerable plaque detection. There are at present three invasive imaging modalities clinically available: intravascular ultrasound (IVUS), near-infrared spectroscopy (NIRS)–IVUS, and optical coherence tomography (OCT). Cumulative data indicate that all three have an indispensable role in guiding intervention, however, histology studies suggest that only OCT and NIRS–IVUS are able to assess plaque characteristics associated with increased vulnerability and detect the presence of necrotic core (NC) tissue—which is an established predictor of adverse events.^1–6^

To facilitate image interpretation, accurate quantification of plaque components and enhance the applications of these two modalities in clinical practice and research, machine learning (ML) methodologies have recently been introduced that allow rapid and reproducible image segmentation and plaque component characterization.^7–11^ These methods differ in their design, depending on the tasks they are aimed at, but also on the characteristics of the processed modality, in the sets used for training and testing, and on their clinical translatability as only a few of them have been incorporated in user-friendly software that allows the processing of large datasets in real-time. Moreover, most of the developed ML methods have been tested against the estimations of experts and not using histology as the reference standard. The aim of the present study is to examine the performance of two clinically available ML methodologies developed to process and characterize plaque composition in NIRS–IVUS and OCT data, using histology as the reference standard.

## Methods

### Matched near-infrared spectroscopy–intravascular ultrasound and histology data

We retrospectively analysed matched NIRSIVUS and histological data obtained from 12 cadaveric hearts collected during the conduction of a study that aimed to test the efficacy of NIRS–IVUS in plaque analysis. The methodology for data collection and matching has been previously described.^1^ Briefly, the hearts were received within 48 h after death; from these, 15 coronary arteries without total occlusions or severe stenoses (minimum lumen area <1 mm) were selected, mounted on a fixture and perfused with human blood at body temperature using a pump that generated pulsatile flow at a rate of 60 cycles/min (coronary blood pressure range: 80–120 mmHg). The blood vessels were interrogated within 96 h post-death by a 3.2F NIRS–IVUS probe that incorporated a 40 MHz IVUS catheter (TVC Imaging System, Infraredx, Bedford, MA, USA) which was first advanced to the distal end of the vessel over a 0.014-inch guidewire and was then pulled back by an automated device at a speed of 0.5 mm/s.

### Matched optical coherence tomography and histology

The OCT dataset consisted of matched OCT and histological data collected in Bristol Heart Bank and Odense University Hospital Svendborg.^12,13^ The Bristol dataset included four autopsied hearts with intact coronary arteries that were excised within 48 h of post-mortem study and stored at 4°C. A guide catheter engaged the ostium of the right coronary artery, and the vessel was fixed with sutures. The specimens were held in a Perspex container that had adapters on both sides of the lid enabling connection of the guide internally, and a Y-connector and a pressure/injector manifold externally. Optical coherence tomography imaging was performed in these vessels using a C7XR Dragonfly (Abbott Vascular, Santa Clara, CA, USA) catheter at a constant speed of 20 mm/s under phosphate buffer saline injection with an intracoronary pressure maintained at 100 mmHg.

The Svendborg dataset was generated to examine the efficacy of computed tomography and OCT imaging in assessing plaque pathology using histology as the reference standard. The dataset consisted of four cadaveric hearts from patients with suspected coronary artery disease who underwent autopsy. The hearts were first rinsed in water, and then the coronary arteries were flushed with water and exposed to a gentle vacuum to remove blood. The ostium of the right and left coronary system was engaged by a sheath and through the sheath saline at 37°C was infused before and during OCT imaging to maintain a coronary artery pressure of 60–80 mmHg; this was achieved by a sphygmomanometer connected to the infusion port. A guide wire was advanced to the studied vessel through the sheath and over the wire, a C7XR Dragonfly catheter was inserted into the distal end of the vessel and pulled back using an automated pullback device at a speed of 20 mm/s. Imaging was performed in the proximal 50 mm of the right and left anterior descending coronary arteries.

### Histological analysis

The vessels imaged with NIRS–IVUS were pressure fixed in formalin and cut at 2 mm intervals; two slides with a thickness of 7 µm were taken at every 2 mm; one was stained with haematoxylin–eosin (H&E) and the other with Russell–Movat’s pentachrome.

The vessels included in the Bristol database were pressure fixed, while they were on the heart, with formalin at 100 mmHg for 15 min, then they were removed from the heart and fixed with formalin for 24 h and finally, they were embedded in paraffin, and cut at every 4 mm. From each of the generated blocks, slices with a thickness of 3 µm were taken that were stained with H&E, Elastic Van Gieson, CD68 for the identification of macrophages, and smooth muscle cell α-actin stains.

Finally, the vessels consisting of the Svendborg database were embedded with Paraffin and sectioned at every 200 µm; from each 200 µm block, sections were obtained with a thickness of 3 µm that were stained with H&E and modified elastic Verhoeff–van Gieson. In a subset, an additional section was obtained that was stained with CD68 to detect the presence of macrophages.

### Intravascular imaging histological analysis

The NIRS–IVUS, OCT, and histological data were matched by two experts (R.B. and C.V.B.) who carefully aligned histological sections with corresponding intravascular imaging frames. Alignment and co-registration were done by comparing the distance of the histological section from the proximal end of the studied vessel with the distance of the intravascular imaging frame (which was acquired at a fixed pullback speed), using the ostium of the vessel or the origin of side branches as landmarks, as well as plaque eccentricity to identify corresponding frames. Only matched frames that were at least ≥0.4 mm apart were included in the present analysis.

In the matched NIRS–IVUS frames, segmentation was performed using a well-validated ML algorithm that enables fully automated detection of the lumen and external elastic membrane (EEM) borders.^14^ The plaque area defined by the annotated borders (EEM area—lumen area) was processed by an ML methodology that takes into account the greyscale pixel intensity in IVUS and the estimations of NIRS for the presence of lipid to classify pixels in three tissue types: fibrotic (FT), calcific (Ca), and lipid core. This classifier has been trained using histological estimations as the reference standard and assumes that the pixels located behind the proximal edge of the calcium in the area where there is acoustic shadowing correspond to Ca.^15^ Both ML methods used for the NIRS–IVUS analysis have been incorporated into the commercial software QCU-CMS (version 4.69, Leiden University Medical Center, Leiden, the Netherlands).

Optical coherence tomography segmentation was performed using a recently presented software developed by Pulse Medical Imaging Technology (Shanghai, China).^9^ The software incorporates a deep convolutional neural network that enables automated detection of the lumen and EEM borders and plaque characterization and identification of FT, Ca, and lipid tissue. In frames where the EEM is not visible in its entire circumference, the software automatically extrapolates the EEM by taking into account the ΕΕμ borders in adjacent frames. The software also allows the detection of cholesterol crystals and macrophages and the measurement of the cap thickness over lipid tissue. The training of the incorporated ML methodology was performed using the estimations of expert analysts from three core labs in >10 000 frames, while its testing was performed in 300 frames, against the estimations of experts as reference standard.

The analysis of the matched histological sections was performed by experienced histopathologists (S.W. and H.P.) in H&E sections blinded to the intravascular image segmentation using dedicated software (Hisedit, version 12). The lumen and EEM were manually annotated, and within the plaque area, the presence of NC (defined as an acellular extracellular lipid pool with evidence of necrosis and cholesterol crystals) and Ca tissue were annotated. When Ca and NC regions overlapped the region was classified as Ca. The remaining plaque was classified as FT—comprised of smooth muscle cells, collagen, and proteoglycans with or without extracellular lipid pools but without necrosis.^16,17^ Classification was restricted to these three tissue types as they are most clinically meaningful. In cases where CD68 staining was available the macrophage accumulation was measured using an in-house fully automated methodology that was incorporated in the Hisedit software.^18^ This approach automatically detects the pixels stained with CD68 that indicate the presence of macrophages and defines macrophages-rich regions as those that have at least 40% concentration in CD68 staining. This cut-off was selected based on a previous histopathological study showing that vulnerable plaques have a macrophage content ≥10% in the entire plaque;^19^ in this analysis a nine-fold higher cut-off was used to define macrophages-rich regions. Macrophage analysis was performed in the entire plaque because the OCT–ML classifier was trained to assess this feature in the entire atheroma and existing evidence supports a prognostic value of this feature when it is measured in the entire plaque and not over fibrous caps where OCT seems to perform better.^2,19–21^

### Examination of intravascular imaging and histological estimations of plaque composition

The analysed histological cross-sections were superimposed onto the corresponding NIRS–IVUS and OCT images and the estimations of histology and ML methodologies for the plaque area, composition, and burden (PB) were compared. In the present analysis, it was assumed that the lipid tissue detected by the OCT–ML and NIRS–IVUS classifiers corresponded to NC identified by histology.

**Figure 1:**
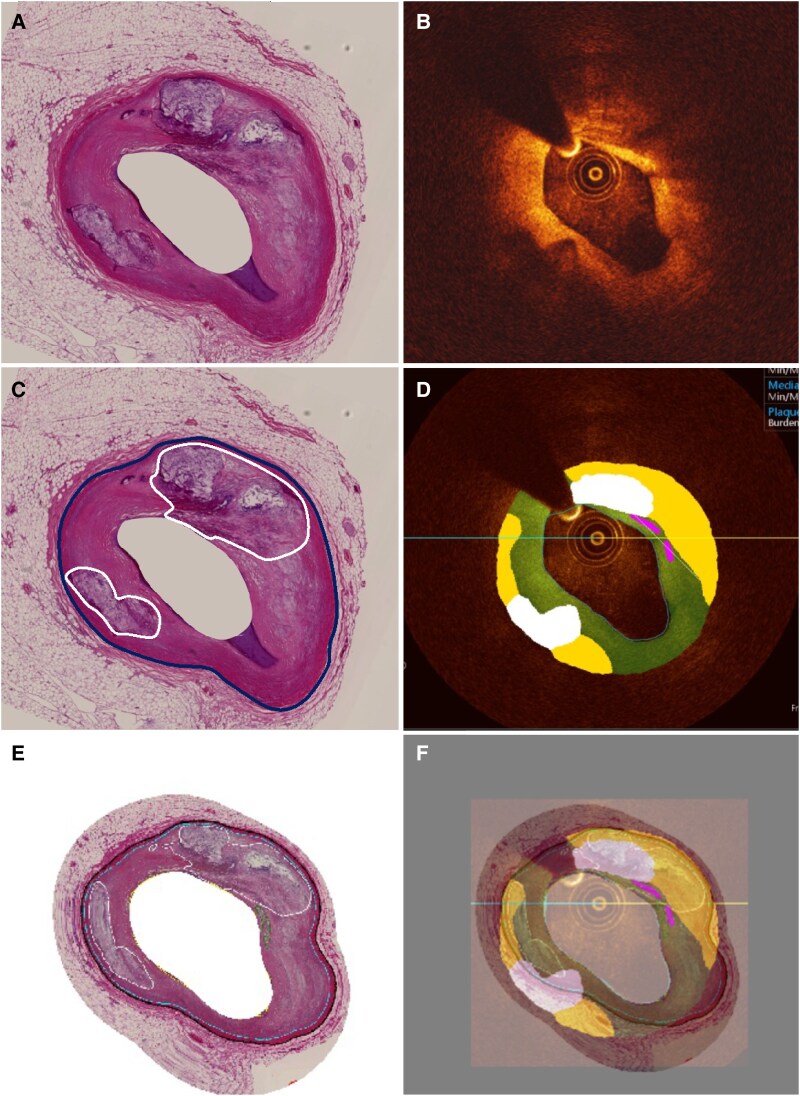
Module developed for co-registering histology and the matched intravascular images. (*A*) A histological section and (*B*) the corresponding optical coherence tomography frame. The annotated histology image is shown in (*C*) with the external elastic membrane and the Ca tissue annotated. (*D*) The output of tissue classification using the OCT–ML classifier. After adjusting the image resolution, using the pixel size information mapping is performed so that the histological image is reshaped to fit the optical coherence tomography frame (*E*). (*F*) The histology and OCT–ML images superimposed on each other to allow overlapping and non-overlapping area analysis of the plaque and tissue types. OCT, optical coherence tomography; ML machine learning.

To optimize co-registration and overcome errors attributed to the different lumen and vessel wall shapes in intravascular imaging data and histology we used an in-house software (*Figure 1*) capable of adjusting the lumen and vessel geometry of the histology images to match the pressurized lumen shape of the intravascular images. This software was built in Matlab (version 2022b) and involves the following steps:

1. Manual delineation of the lumen borders in corresponding intravascular imaging and histological sections.
2. Manual identification of corresponding points in these borders; the correspondence is based on the origin of side branches and on the circumferential distribution of the plaque. In this step, there is an assumption that there is a correct annotation of corresponding points between the lumen border in intravascular imaging and histological data. This step was done by two experts (R.B. and R.P.), with final checking and refinement as needed by a 3rd expert (CVB).
3. Histological image ‘strips’ are taken (outward) along the lumen contour, with a user-specified strip thickness.
4. The histological image strip is placed along the lumen border in the corresponding NIRS–IVUS or OCT frames. The border is scaled circumferentially in reference to the corresponding points from Step 2, and also scaled radially based on the difference of resolution (pixel size) between histology and NIRS–IVUS/OCT frames. In this step, an assumption is made that the plaque area is not compressed or elongated after transformation.

The output of this process is a hybrid histological and NIRS–IVUS/OCT image that enables accurate examination of the estimations of intravascular imaging and histology for the plaque composition and distribution.

Two types of analyses were performed between intravascular imaging and histological estimations. The first was a quantitative analysis which included examination of the estimations of the two ML approaches and histology for EEM, plaque area (defined as EEM–lumen area), and PB (plaque area/EEM area × 100) and FT, Ca, and NC areas. Lumen areas are not compared as tissue shrinkage in decompressed histological sections cannot be validly compared with intravascular images acquired in pressurized vessels.

The second analysis was a qualitative analysis and involved a morphological comparison of the plaque distribution derived by the ML methodologies and histology. More specifically we estimated the proportion of plaque area where intravascular imaging and histological estimations overlap—overlapping area analysis—as well as the area where these two estimations did not overlap—non-overlapping area analysis. In addition, we examined the estimations of ML and histology for plaque composition using the following metrics:

- Region-level analysis: in this analysis, the estimations of histology were used to define regions of each plaque component (regions of interest, ROI) and in each ROI the predominant tissue type, defined as the plaque component with the largest proportion, derived by NIRS–IVUS–ML or OCT–ML was reported.
- Area-level analysis: this involves the computation of the areas of different plaque components—derived by NIRS–IVUS–ML or OCT–ML—that overlap with the ROIs defined by histology. The proportion of the correct estimation was reported using the histological classification as the reference standard.

### Statistical analysis

Continuous variables are presented as mean and standard deviation and categorical as absolute values and percentages. Data distribution was tested using the Kolmogorov–Smirnov test and the normally distributed variables were compared using the Student’s *t*-test, whereas the Mann–Whitney U test was used to compare non-normally distributed variables.

Mann–Whitney U analysis and Bland–Altman analysis were used to compare the estimations of intravascular imaging and histology for the EEM area, plaque area and PB, and FT, Ca, and NC areas. The concordance correlation coefficient (CCC) was used to examine the agreement between intravascular imaging and histological estimations.

The performance of the NIRS–IVUS–ML and OCT–ML classifiers in characterizing plaque composition at the region- and area level was assessed using sensitivity, precision, *F*-score, and the overall accuracy of the tested method. Sensitivity was calculated by the ratio: true positive/(true positive + false negative), precision using the ratio: true positive/(true positive + false positive), and *F*-score by calculating: 2 × (precision × sensitivity)/(precision + sensitivity). Finally, the overall accuracy represented the number of the correct estimations of the ML classifiers over the total estimations.

A *P*-value <0.05 was considered statistically significant. Analyses were performed using SPSS version 23 (IBM, Armonk, New York, NY, USA) and MedCalc software version 19.1.6 (Ostend, Belgium).

## Results

### Studied patients

In total, 20 vessels from 20 patients were included in the present analysis. Matched NIRS–IVUS and histological sections were available in 12 vessels (12 patients), and OCT and histological sections in eight vessels [eight patients; four vessels from the Svendborg (four patients) and four (four patients) from the Bristol dataset]. The mean age of the NIRS–IVUS cohort was 60 ± 9 years while, the mean age of the OCT group 68 ± 13 years. The baseline demographics of the patients included in the two cohorts are shown in Supplementary material online, *Table S1*.

In the NIRS–IVUS cohort, 131 NIRS–IVUS and histological frames were matched and included in the analysis. In the OCT cohort, matching was possible for 219 frames; from these 35 frames were excluded because the OCT–ML classifier did not allow accurate delineation of the lumen border (*n* = 7), or because the entire plaque was not visible in OCT or due to image artefacts (*n* = 28); therefore, 184 matched cross section were included in the final analysis (see Supplementary material online, *Figure S1*).

### Atheroma burden and tissue distribution in histological datasets

The PB in the NIRS–IVUS cohort was smaller compared with the OCT cohort but there was no difference between groups in the plaque area (see Supplementary material online, *Table S2*). Necrotic core was detected in 33.6% and Ca in 39.7% of the histological sections in the NIRS–IVUS cohort while in the OCT dataset, the incidence of these two tissue types was 40.8% and 39.1%, respectively. There was no significant difference between groups for FT, Ca, and NC areas (*Table 1*).

**Table 1:** Quantitative analysis of plaque area and tissue component detection between histology and the respective NIRS–IVUS–ML and OCT–ML datasets

	Histology	NIRS–IVUS–ML	Mean difference	P*	CCC (95% CI)	P**	Histology	OCT–ML	Mean difference	P*	CCC (95% CI)	P**	
EEM area, mm^2^	13.43 ± 4.89	14.94 ± 5.37	−1.51 ± 1.56	0.012	0.91 (0.89–0.94)	<0.001	11.53 ± 5.01	9.79 ± 4.96	1.72 ± 1.53	<0.001	0.90 (0.87–0.92)	<0.001	
Plaque area, mm^2^	7.03 ± 3.06	8.53 ± 3.64	−1.51 ± 1.56	<0.001	0.81 (0.75–0.86)	<0.001	7.00 ± 2.66	5.28 ± 2.18	1.72 ± 1.53	<0.001	0.64 (0.57–0.70)	<0.001	
Plaque burden, %	51.92 ± 12.20	56.4 ± 12.2	−4.46 ± 4.23	0.004	0.88 (0.84–0.91)	<0.001	63.89 ± 11.71	56.18 ± 10.70	7.70 ± 4.80	<0.001	0.73 (0.68–0.78)	<0.001	
FT area, mm^2^	6.08 ± 2.48	6.78 ± 3.14	−0.70 ± 2.01	0.107	0.73 (0.64–0.79)	<0.001	6.03 ± 2.15	4.02 ± 1.73	2.02 ± 1.67	<0.001	0.42 (0.33–0.49)	<0.001	
Ca area, mm^2^	0.73 ± 1.50	1.35 ± 2.65	−0.61 ± 1.42	0.351	0.75 (0.71–0.80)	<0.001	0.73 ± 1.19	0.37 ± 0.77	0.37 ± 0.82	0.011	0.62 (0.55–0.69)	<0.001	
NC area, mm^2^	0.22 ± 0.67	0.47 ± 1.30	−0.25 ± 0.83	0.389	0.66 (0.59–0.72)	<0.001	0.23 ± 0.40	0.73 ± 1.17	−0.60 ± 1.10	<0.001	0.13 (0.08–0.17)	0.079	

CCC, concordance correlation coefficient; ML, machine learning; NIRS–IVUS, near-infrared spectroscopy intravascular ultrasound; OCT, optical coherence tomography.

P* indicates statistical significance for the Mann–Whitney U analysis between intravascular imaging and histology and P** denotes statistical significance for the CCC.

### Near-infrared spectroscopy–intravascular ultrasound–machine learning compared with histology

#### Quantitative analysis: plaque and tissue component areas

**Figure 2:**
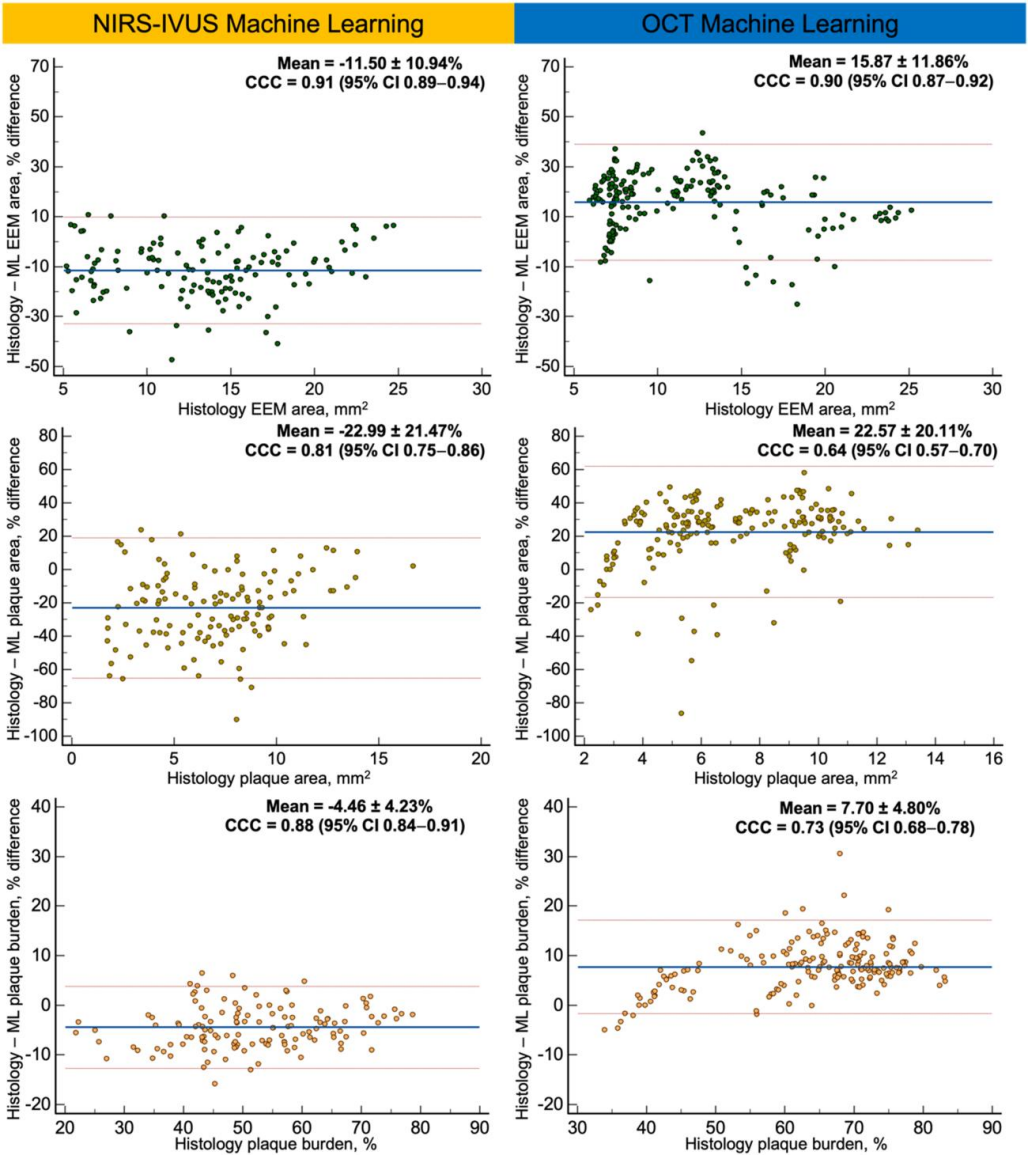
Bland–Altman analyses comparing NIRS–IVUS–ML estimations with histology and OCT–ML with histology for external elastic membrane, plaque area, and plaque burden. NIRS, near-infrared spectroscopy; IVUS, intravascular ultrasound; OCT, optical coherence tomography; ML machine learning.

*Table 1* and *Figure 2* show the estimations of histology and of NIRS–IVUS–ML and OCT–ML for the EEM area, plaque area, PB, and plaque components. Mann–Whitney U analysis showed that the estimations of NIRS–IVUS–ML differed from those of histology for EEM, plaque area, and PB. NIRS–IVUS–ML overestimated EEM area, plaque area, and PB in comparison with histology. A strong correlation assessed by CCC was noted for the EEM area, plaque area, and PB.

**Figure 3:**
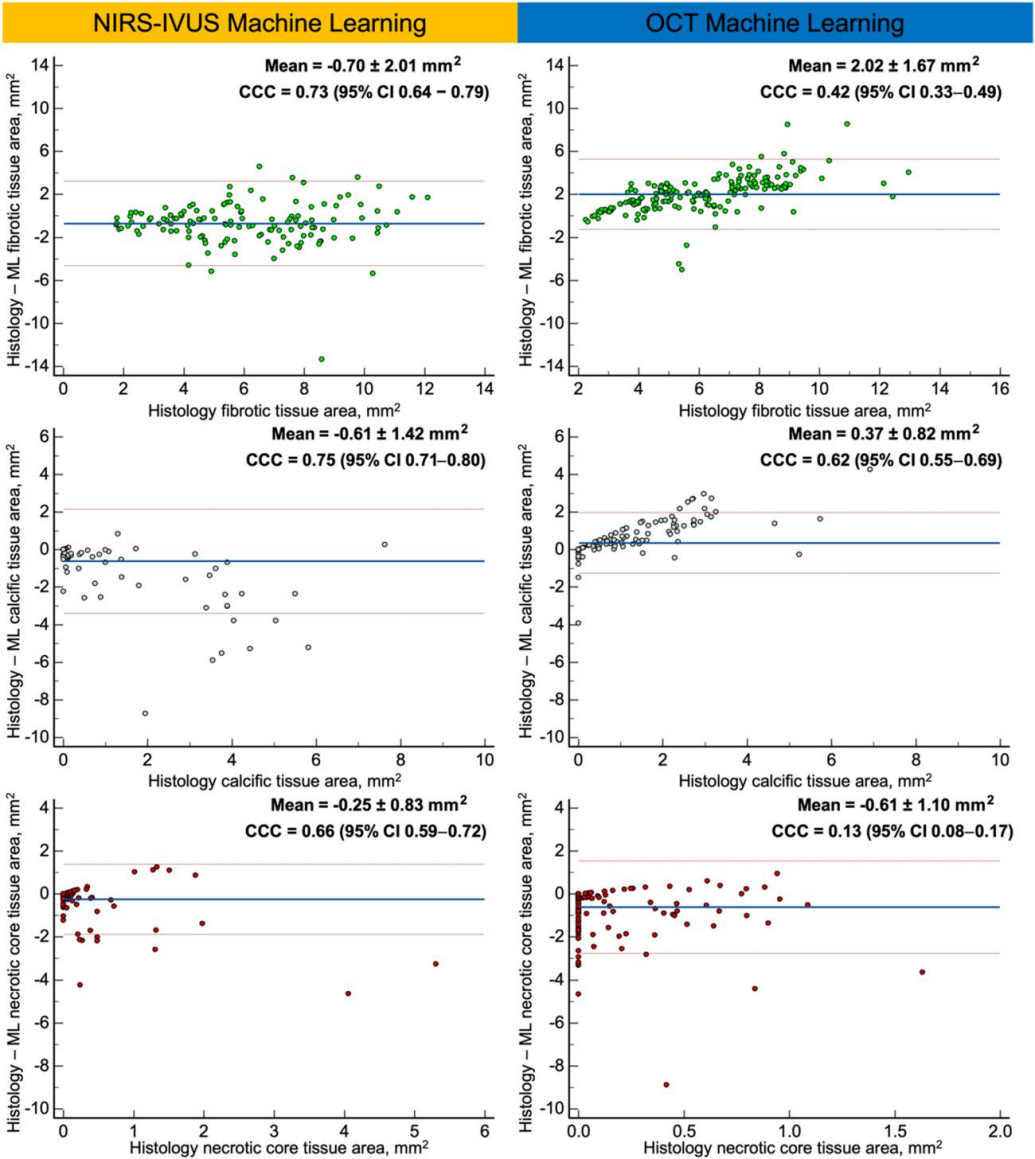
Bland–Altman analyses comparing NIRS–IVUS–ML with histology and OCT–ML with histology for fibrotic, calcific, and necrotic core tissue. NIRS, near-infrared spectroscopy; IVUS, intravascular ultrasound; OCT, optical coherence tomography; ML machine learning.

Tissue component areas estimated by NIRS–IVUS–ML were not significantly different from histology, but Bland–Altman analysis showed that NIRS–IVUS–ML biased towards overestimating FT, Ca, and NC areas compared with histology (*Figure 3*). Concordance correlation coefficient demonstrated a moderate concordance between NIRS–IVUS–ML and histology for the NC area (0.66) and strong for the FT and Ca areas (0.73 and 0.75, respectively).

#### Qualitative analysis: plaque area detection

Overlapping area analysis showed a high agreement for plaque area detection between histology/NIRS–IVUS–ML (93.9%, see Supplementary material online, *Table S3*). NIRS–IVUS–ML tended to overestimate the plaque area compared with histology, with no significant correlation found for PB and degree of NIRS–IVUS–ML misclassification. The presence of calcific tissue was associated with an overestimation of the plaque area; conversely, the NC burden did not appear to affect the performance of NIRS–IVUS–ML (see Supplementary material online, *Figure S2*).

#### Qualitative analysis: plaque component distribution

None of the tissue-type ROIs were beyond the borders of the EEM detected by NIRS–IVUS–ML. We found that NIRS–IVUS–ML had a high sensitivity, precision, and *F*-score for detecting FT and Ca ROIs, whereas for the NC the modality had high precision (0.89) but lower sensitivity (0.43) and *F*-score (0.58) (*Table 2*). Overall performance showed excellent sensitivity, precision, and recall with an accuracy of 83% for regional classification. Area-level analysis demonstrated high sensitivity, precision, and *F*-score for FT; moderate sensitivity and *F*-score and high precision for Ca; and moderate precision for NC but low sensitivity and *F*-score. The overall area-level performance was high with an accuracy of 89%. Overall region and area-level classification performance is shown in Supplementary material online, *Figures S3* and *S4*.

**Table 2:** Confusion matrices showing the performance of the NIRS–IVUS–ML in assessing tissue component distribution at a region and area level using histology estimations as a reference standard

	NIRS–IVUS–ML classification			Validation metrics			
	Fibrotic	Calcific	Necrotic core	Sensitivity	Precision	*F*-score	Accuracy (%)
Histology classification							
*Region-level analysis*							
Fibrotic regions	140	10	3	0.92	0.82	0.86	—
Calcific regions	2	64	0	0.97	0.82	0.89	—
Necrotic core regions	29	4	25	0.43	0.89	0.58	—
Overall performance	—	—	—	**0**.**83**	**0**.**83**	**0**.**81**	**82.67**
*Area-level analysis*							
Fibrotic tissue, mm^2^	316.13	2.28	6.06	0.97	0.91	0.94	—
Calcific tissue, mm^2^	11.50	25.38	1.51	0.66	0.89	0.76	—
Necrotic core tissue, mm^2^	20.77	0.84	11.53	0.35	0.60	0.44	—
Overall performance	—	—	—	**0**.**89**	**0**.**88**	**0**.**88**	**89.15**

Overall performance of Sensitivity, Precision and F-score in bold represent the weighted average of each taking into account the relative proportion of each region or tissue area in the analysis.

ML, machine learning; NIRS–IVUS, near-infrared spectroscopy intravascular ultrasound.

### Optical coherence tomography–machine learning compared with histology

#### Quantitative analysis: plaque and tissue component areas

Like NIRS–IVUS–ML, OCT–ML was found to differ with the histological estimations for the EEM area, plaque area, and PB but in contrast with NIRS–IVUS–ML, OCT–ML was found to consistently underestimate these metrics (*Table 1* and *Figure 2*). OCT–ML had a larger bias and limits of agreement compared with histology for the PB (underestimation 7.70 ± 4.80%). While a strong correlation was noted for the EEM area (CCC: 0.90), the agreement was lower for the plaque area (CCC: 0.64), and burden (CCC: 0.73).

Tissue compositional analysis showed that the OCT–ML estimations for the areas of the three plaque components areas were significantly different from those of histology. Bland–Altman analysis (*Figure 3*) demonstrated that the OCT–ML underestimated FT and Ca areas but overestimated NC areas. OCT–ML was found to have a moderate CCC (0.62) with histology for Ca and FT (0.62 and 0.42, respectively) and weak CCC for NC area (0.13).

#### Qualitative analysis: plaque area detection

Overlapping area analysis showed that the OCT–ML detected a significantly lower plaque area in matched histology compared with the NIRS–IVUS–ML (86.7% vs. 93.9%; *P* = 0.040, Supplementary material online, *Table S3*). The non-overlapping areas between histology/NIRS–IVUS–ML and histology/OCT–ML were similar. However, the source of error was different; in contrast to NIRS–IVUS–ML, OCT–ML underestimated the plaque area compared with histology, and the underestimation error in OCT–ML was weakly correlated with increasing PB (*r* = 0.30, *P* < 0.001). As with NIRS–IVUS–ML, the presence of Ca tissue was associated with an overestimation of the plaque area while NC burden did not appear to impact its performance (see Supplementary material online, *Figure S2*).

#### Qualitative analysis: plaque component distribution

The fact that the OCT–ML classifier underestimated the plaque areas in heavily diseased segments impacted the efficacy of the modality in assessing plaque components. More specifically, 3.68 mm^2^ (17%) of the NC area and 30.98 mm^2^ (34%) of the Ca area detected by histology were outside the ML-estimated EEM borders and therefore were missed by the OCT–ML approach.

When analysis was restricted to areas with an overlap between the plaque estimations of histology and intravascular imaging, we found that OCT–ML had a high sensitivity, precision, and *F*-score for the FT ROIs and a moderate sensitivity and *F*-score but an excellent precision for the Ca regions (*Table 3*). For the NC regions, the OCT–ML had a weak sensitivity (0.25), precision (0.29), and *F*-score (0.27). Overall, the OCT–ML had a high moderate sensitivity, precision, and *F*-score for regional analysis with an accuracy of 72%. Area-level analysis showed similar performance for FT, lower sensitivity, precision, and *F*-score for Ca and poor sensitivity precision and *F*-score for NC area classification. The OCT–ML had an overall accuracy of 78%. The region and area-level classification performance are shown in Supplementary material online, *Figures S3* and *S4*.

**Table 3:** Confusion matrices showing the performance of the OCT–ML in assessing tissue component distribution at a region and area level using histology estimations as a reference standard

	OCT–ML classification			Validation metrics			
	Fibrotic	Calcific	Necrotic core	Sensitivity	Precision	*F*-score	Accuracy (%)
Histology classification							
*Region-level analysis*							
Fibrotic regions	165	0	19	0.90	0.75	0.82	—
Calcific regions	14	52	15	0.64	0.98	0.78	—
Necrotic core regions	41	1	14	0.25	0.29	0.27	—
Overall performance	—	—	—	**0**.**72**	**0**.**68**	**0**.**77**	**71.96**
*Area-level analysis*							
Fibrotic tissue, mm^2^	501.62	8.97	95.45	0.83	0.94	0.88	—
Calcific tissue, mm^2^	19.02	27.40	12.60	0.46	0.75	0.57	—
Necrotic core tissue, mm^2^	12.05	0.00	6.16	0.34	0.05	0.09	—
Overall performance	—	—	—	**0**.**78**	**0**.**92**	**0**.**65**	**78.33**

Overall performance of Sensitivity, Precision and F-score in bold represent the weighted average of each taking into account the relative proportion of each region or tissue area in the analysis.

ML, machine learning; NC, necrotic core; OCT, optical coherence tomography.

#### Macrophage detection by optical coherence tomography

In total 67 histological sections were stained with CD68 and matched with OCT frames. Fourteen of these sections did not have any macrophages (see Supplementary material online, *Table S4*), the OCT–ML failed to identify macrophages in 46/53 (87%) sections while in the remaining sections, the OCT–ML classifier significantly underestimated their extent. The total macrophage-rich region in histology was 53.73 and 2.21 mm^2^ in the OCT–ML images. There was no concordance between OCT–ML and histology for macrophage-rich regions (CCC: 0.02, *P* = 0.872).

## Discussion

The present study examined the performance of two clinically available ML methodologies for automated quantification and characterization of PB and composition against a histological standard. Analysis was done in a large set of matched histological and intravascular imaging data using an efficient methodology to co-register corresponding sections. We found that both ML approaches provided good estimations about plaque area and PB with NIRS–IVUS–ML being more accurate overall, as OCT–ML tended to underestimate plaque area, especially in diseased segments. Plaque compositional analysis demonstrated that the NIRS–IVUS–ML-enabled accurate quantification of the FT, Ca, and NC areas and provided accurate estimations for the FT and Ca tissue distribution while the method had a moderate performance for the NC tissue. Conversely, the OCT–ML approach underestimated FT and overestimated NC areas, and whilst it appeared capable of estimating FT and Ca tissue distribution, it had a weak performance for NC tissue. Additionally, the OCT–ML was unable to identify the presence of macrophages.

Conventional intravascular image analysis is currently performed by experts—however, this is an inefficient and time-consuming process prone to human bias, while its reproducibility depends heavily on individuals’ expertise.^22,23^ Several reports have demonstrated that human experts have limited performance in characterizing plaque phenotypes and the distribution of plaque components, highlighting the need for automated methodologies to deliver reliable and rapid analysis at scale.^24,25^

Approaches that have been presented in the literature for IVUS-based plaque characterization have been proven inefficacious when tested against histology.^5,26–30^ To overcome this limitation, the fusion of NIRS with IVUS imaging has been proposed. Cumulative data have shown that this approach is superior to standalone IVUS for detecting plaque phenotypes—however, it does not allow quantification of tissue types and precise localization of the lipid tissue within the plaque. To address this drawback, an ML classifier has recently been designed that was trained using histology and takes advantage of the information provided by both IVUS and NIRS to assess tissue component distribution.^15^

Additionally, methods have been developed for OCT-based plaque characterization that relied either on the analysis of the backscattered signal^31–55^ or on ML algorithms trained either by experts,^34,35^ or histology.^10,11,36,37^ The studies that used histology for the training of ML classifiers have significant limitations, as they included small numbers of histological sections, were unable to provide predictions of the EEM border in frames where these were not visible, and have not been incorporated in user-friendly platforms that will allow processing of large datasets. These have been overcome by the methodology introduced by Chu *et al.*^34^ which took advantage of the annotations of expert analysts from established imaging core labs to train a methodology for the quantification of the PB and its composition in OCT. This approach has been used in research to examine the implications of endovascular devices on plaque composition and stratify cardiovascular risk, and in a recent report the ratio of the fibrous cap thickness vs. the NC burden was found to be a strong predictor of future events.^38,39^ However, despite the broadening use of this approach, no robust validation of its efficacy has yet been reported using a histological standard.

The present study was designed to address this need and examine the performance of different ML approaches for NIRS–IVUS and OCT plaque compositional analysis against a histological standard. We found that NIRS–IVUS–ML allows more accurate detection of the plaque area than OCT–ML, albeit with a tendency to overestimate the plaque area, while the latter tended to underestimate it. The overestimation of the plaque area by the NIRS–IVUS–ML is likely to be due to tissue shrinkage during histological preparation but also to the fact that IVUS overrates lumen and EEM dimensions as shown in previous reports;^40,41^ conversely, OCT may provide more accurate estimations of the lumen’s dimensions than IVUS, but it often fails to assess the entire plaque because of its poor penetration depth. It is apparent that the extrapolation of EEM by the OCT–ML methodology using adjacent frames failed to provide reliable estimations of the PB in this dataset (see Supplementary material online, *Figure S5*). These findings appear to contradict a recently published study^42^ which showed that the OCT–ML reported a diagnostic accuracy of 92% in identifying frames with a PB >65%. However, the latter study included a small number of frames (*n* = 64, with only six frames with a PB >65%) and used IVUS as a reference standard rather than histology.

Quantitative analysis focusing on plaque components demonstrated that NIRS–IVUS–ML overestimated FT, Ca, and NC tissue areas compared with histology which can attributed to the known tendency of IVUS to overestimate the plaque area compared with histology.^43–45^ We also found the NIRS–IVUS–ML was accurate in assessing FT distribution, had a moderate accuracy for the Ca tissue distribution and a weaker performance for the NC tissue distribution—misclassifying 63% of NC areas as FT achieving a sensitivity of 35% for NC areas. The performance of NIRS–IVUS–ML for the detection of histologically defined NC can be explained by the fact that NIRS is able to detect lipid tissue which may not necessarily equate to NC as shown in previous histopathological studies,^46–48^ while in Ca areas the modality is limited in visualizing the distal edge of Ca because of shadowing artefact leading to overestimation of Ca tissue and low specificity.

**Figure 4:**
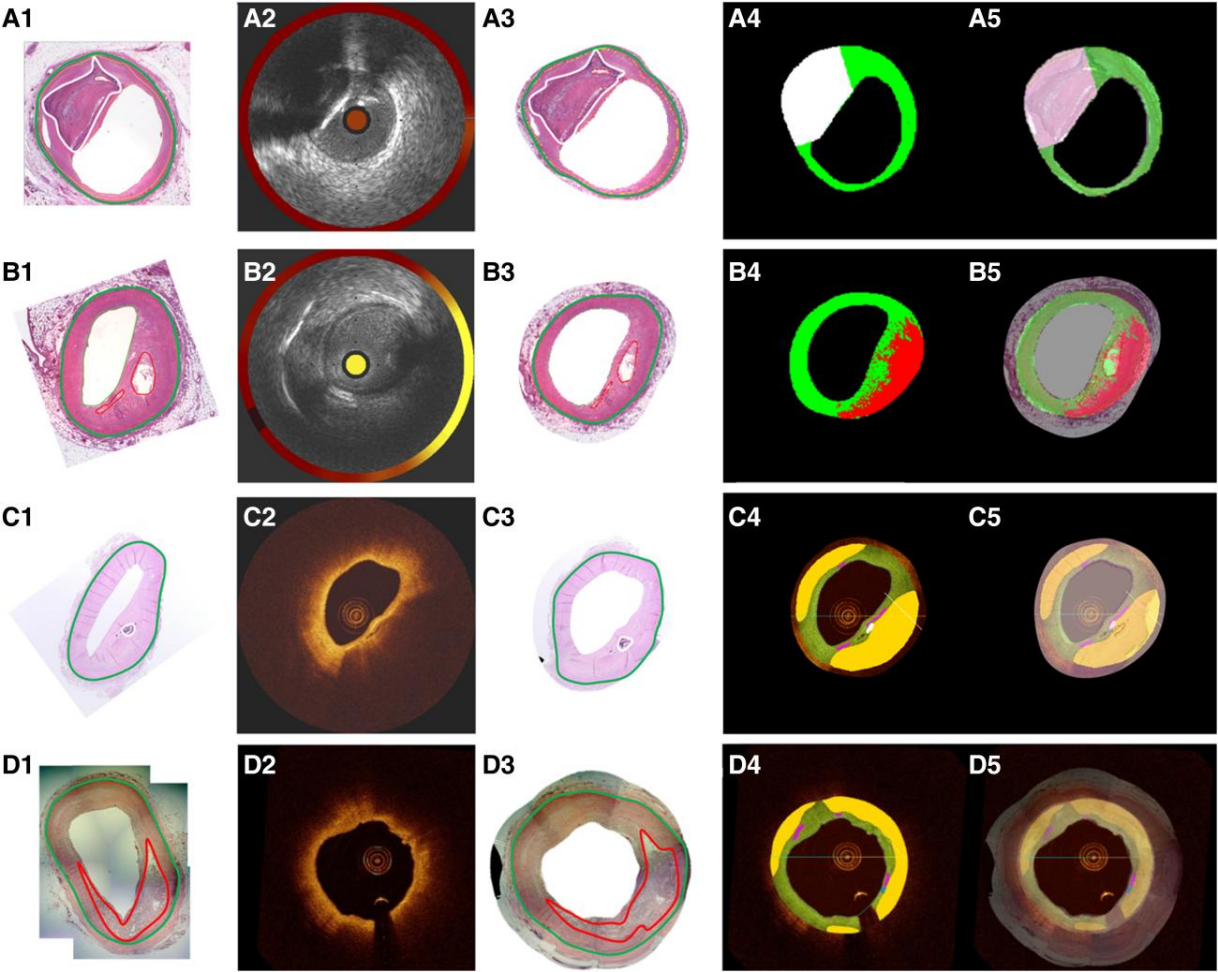
Representative examples of plaque component classification performance by the NIRS–IVUS–ML and OCT–ML classifiers. (*A*1) A histological section with calcific plaque annotated in white and the external elastic membrane border annotated in green, the corresponding matched NIRS–IVUS frame is seen in (*A*2), the histology section remapped to the shape of the pressurized intravascular image is shown in (*A*3), the output of the NIRS–IVUS–ML classifier is shown in (*A*4) (green pixels are classified as fibrotic, white as calcific and red as necrotic core) and the overlay of the histology with the ML output for classification performance is shown in (*A*5). (*B*1–*B*5) An example of fibroatheroma and NIRS–IVUS–ML. (*C*1–*C*5) A calcific plaque, and (*D*1–*D*5) a fibroatheroma with corresponding OCT–ML classifier outputs—the OCT–ML classifier output in *C*4 and *D*4 classifies green pixels for fibrotic, white for calcific, yellow for lipid and purple for macrophages. NIRS, near-infrared spectroscopy; IVUS, intravascular ultrasound; OCT, optical coherence tomography; ML machine learning.

Validation of the OCT–ML demonstrated that this classifier tended to quantitatively underestimate the FT and Ca areas and overestimate the NC areas. Qualitative validation demonstrated a high weighted precision of the OCT–ML for the area-level analysis that was similar to NIRS–IVUS–ML. However, as *Table 3* indicates this approach had limited efficacy in detecting NC accurately while it performed well for the FT and Ca tissue. Region-level analysis showed a good performance of the OCT–ML classifier for FT, moderate performance for Ca, and a weak performance for NC regions. These findings should be attributed to the fact that the algorithm misclassified 66% of NC tissue as FT; this error was at least partially caused by the interpolation of the EEM in diseased segments, where the limited penetration depth of OCT would not typically allow visualization of tissue types in the distal plaque. In these areas, there is increased signal attenuation, and consequently, the algorithm classified all the deeply embedded tissues as NC (*Figure 4*) resulting in an overestimation of the NC burden (see Supplementary material online, *Figure S6*). These approximations as well as the fact that the OCT–ML method was trained using the estimations of human analysts who have limited efficacy in detecting NC tissue, especially when this is deeply embedded, have influenced the reported results.^25,49,50^ Finally, we demonstrated that OCT–ML does not allow accurate detection of the presence of macrophages—a finding that is supported by previous reports examining macrophage distribution in the entire plaque.^51^

The findings of this report underscore the limitations of existing clinically available ML approaches for plaque characterization in intravascular imaging data and emphasize the importance of developing ML approaches that will be trained and tested in large histology datasets. This is necessary to improve diagnostic accuracy and unlock the full potential of standalone (IVUS or OCT) and hybrid intravascular imaging catheters in characterizing plaque morphology. Prospective studies in large histology datasets assessed by different intravascular imaging catheters are warranted to formally compare the performance of different imaging modalities in assessing plaque pathology.

### Limitations

There are several limitations of this report which should be acknowledged. Even though the number of matched frames included was large, the amount of Ca and NC components was limited, preventing a more thorough evaluation of the performance of the two classifiers. Additionally, this study utilized three histological datasets collected *ex vivo* under different pressure conditions. It has been shown that the pressure applied to the vessel during intravascular imaging can affect plaque area measurements; however, this effect is minor^52,53^ and it is unlikely to have affected the reported results. Moreover, histological analysis was performed by three different histology core labs and this may have some impact in the performance reported by the ML approaches for plaque component classification, but it is unlikely to have influenced the reported findings. Although frame-by-frame matching of intravascular imaging and histology data was done scrupulously, errors in the co-registration of these datasets may have occurred. Additionally, the rotational orientation of matched NIRS–IVUS/OCT and histology frames was done by experts. This process was heavily dependent on the experts’ visual estimation; for this reason, this step was done by three experts, and the final decision was reached by consensus. The superimposition of histology and intravascular imaging was done using a dedicated in-house software which allowed compensation for the impact of different lumen shapes between depressurized histological sections and intravascular imaging, but it was unable to overcome the tissue shrinkage that may occur during histological preparation. This may have resulted in suboptimal co-registration and underestimation of the performance of both NIRS–IVUS–ML and OCT–ML. In this study, H&E was used to assess tissue types in histology. Whilst additional staining with Oil-Red-O or Sudan could potentially add additional confidence for the detection of lipid, these also have limited specificity and are limited by the histological processing methods used in these datasets. The surrogate marker of white ‘empty’ spaces in paraffin-embedded formalin-fixed tissue removed during processing used in this study has been highly correlated with the presence of extracellular lipid.^54^ Moreover, it can be argued that both NIRS and OCT are not specific in detecting NC—NIRS can identify the biochemical composition of lipid tissue and lights up in the presence of macrophages^55^—and OCT signal tends to attenuate in both early lipid pools without necrosis as well as in NC and in the presence of macrophages. Acknowledging the inability of both modalities to differentiate lipid pools from NC, we decided in this analysis to focus on the detection of the NC as this tissue type has been associated with increased plaque vulnerability and worse outcomes. Finally, it should be stressed that this study does not enable a direct comparison of the performance of the developed ML methods or of NIRS–IVUS and OCT in assessing plaque types as validation was performed in different datasets that were acquired and analysed at different time points. Nevertheless, this report is important as it provides a detailed validation of two commercially available ML approaches for plaque characterization, highlights their potential advantages and limitations, and underscores the need to standardize protocols for testing future ML solutions, that will allow comprehensive assessment of their performance before these being applied in the clinical practice and research.

## Conclusions

Machine learning-based analysis of multi-modality NIRS–IVUS data has a moderate efficacy in characterizing plaque composition while the OCT-based plaque characterization using commercially available ML solution had a weak efficacy in detecting NC tissue. This may be attributed to the limitations of standalone intravascular imaging for plaque compositional analysis, as well as the reference standard used for training the OCT–ML classifier being human experts and not a histological standard.

## Supplementary Material

ztaf009_Supplementary_Data

## Data Availability

The data underlying this article were provided by Infraredx, Bristol Heart Bank, and Odense University Hospital Svendborg by permission. Data will be shared on request to the corresponding author only if the supplying institutions agree to grant permission.
