# Supplementary material for: Examination of the performance of machine learning-based automated coronary plaque characterization by near-infrared spectroscopy–intravascular ultrasound and optical coherence tomography with histology
Source: Eur Heart J Digit Health. 2025 Mar 4;6(3):359–71. doi: 10.1093/ehjdh/ztaf009 (PMC12088723; doi:10.1093/ehjdh/ztaf009)
Supplement: ztaf009_Supplementary_Data [file ztaf009_supplementary_data.zip › Supplementary file.docx]

**Examination of the performance of machine learning-based automated coronary plaque characterization by NIRS-IVUS and OCT with histology – Data Supplement**

Retesh Bajaj^1,2,3,#^, Ramya Parasa^1,2,4,#^, Alexander Broersen^5^, Thomas Johnson^6^, Mohil Garg^7^, Francesco Prati^8,9,^ , Murat Çap^1^, Nathan Angelo Lecaros Yap^2^, Medeni Karaduman^10^; Carol Ann Glorioso Rexen Busk^15, 16, 17^, Stephanie Grainger^11^, Steven White^12^, Anthony Mathur^1,2^, Hector M García-García^7^, Jouke Dijkstra^13^, Ryo Torii^14^, Andreas Baumbach^1,2^, Helle Precht^15, 16, 17,*^, Christos V Bourantas^1,2,*^

^1^ Department of Cardiology, Barts Heart Centre, Barts Health NHS Trust, London, UK

^2^ Centre for Cardiovascular Medicine and Device Innovation, William Harvey Research Institute, Queen Mary University of London, UK

^3^ Ottawa Heart Institute, Ontario, Canada

^4^ The Essex Cardiothoracic Centre, Basildon, UK

^5^ Division of Image Processing, Department of Radiology, Leiden University Medical Center, Leiden, The Netherlands

^6^ University Hospitals Bristol and Weston NHS Foundation Trust, Bristol, UK

^7^ Department of Cardiology, Medstar Cardiovascular Research Network, Medstar Washington Hospital Center, Washington, District of Columbia.

^8^ Cardiovascular Sciences Department, Interventional Cardiology Unit, San Giovanni Addolorata Hospital, Rome, Italy

^9^ Centro per la Lotta Contro L’Infarto - CLI Foundation, Rome, Italy

^10^ Department of Cardiology, Faculty of Medicine Yuzuncu Yil University Van, Turkey

^11^ Infraredx, Bedford, MA, United States

^12^ Biosciences Institute, Newcastle University, UK

^13^ Department of Radiology, Leiden University Medical Center, Leiden, The Netherlands

^14^ Department of Mechanical Engineering, University College London, London, UK

^15^ Health Sciences Research Centre, UCL University College, Odense, Denmark

^16^ Department of Radiology, Lillebaelt Hospital, University Hospitals of Southern Denmark, Denmark

^17^ Department of Regional Health Research, University of Southern Denmark, Odense, Denmark

^#^ The first two authors contributed equally to this work.

**^*^Address for correspondence**

**
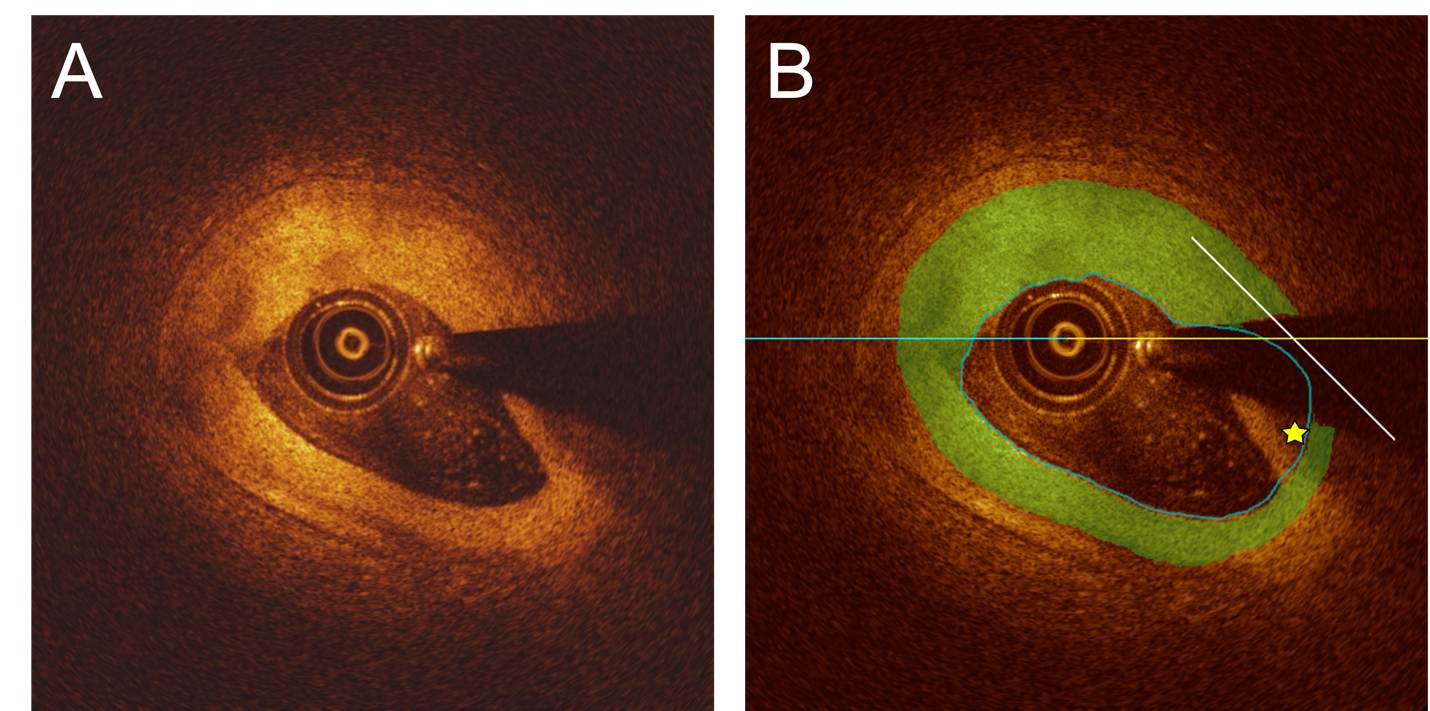
**

**Supplementary Figure 1.** Representative example of erroneous automated lumen detection: the OCT frame (A) and the corresponding ML-based plaque detection and characterization (B) output are shown. There is a misclassification of the lumen and corresponding plaque at 4 o’clock due to tangential signal drop-out artifact (yellow star).

**
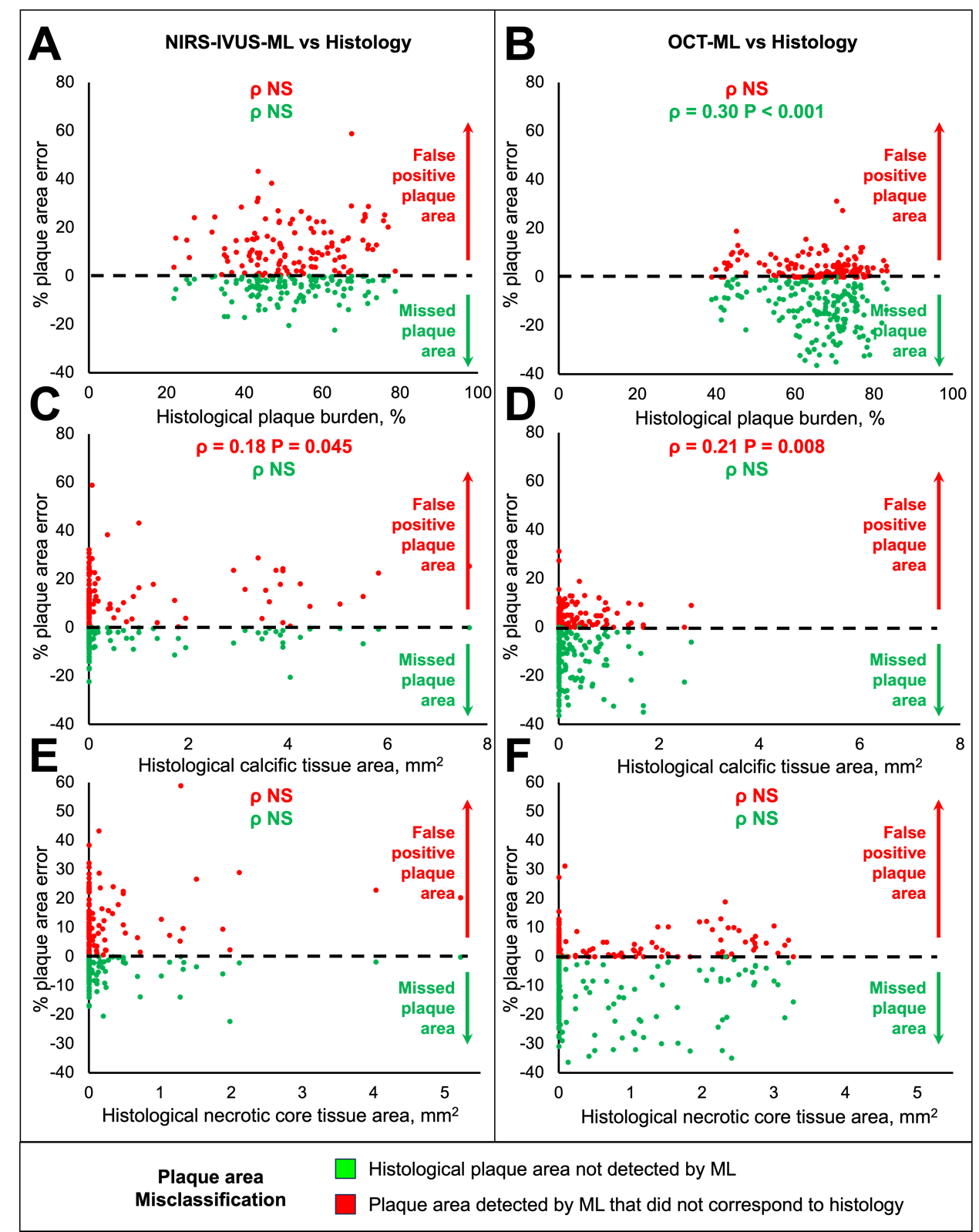
**

**Supplementary Figure 2.** Non-overlapping area analysis demonstrating misclassification errors of the NIRS-IVUS-ML and OCT-ML derived plaque areas compared to histological plaque area, and relationship of misclassification with plaque burden (A, B), calcific tissue area (C, D) and necrotic core tissue area (E, F).


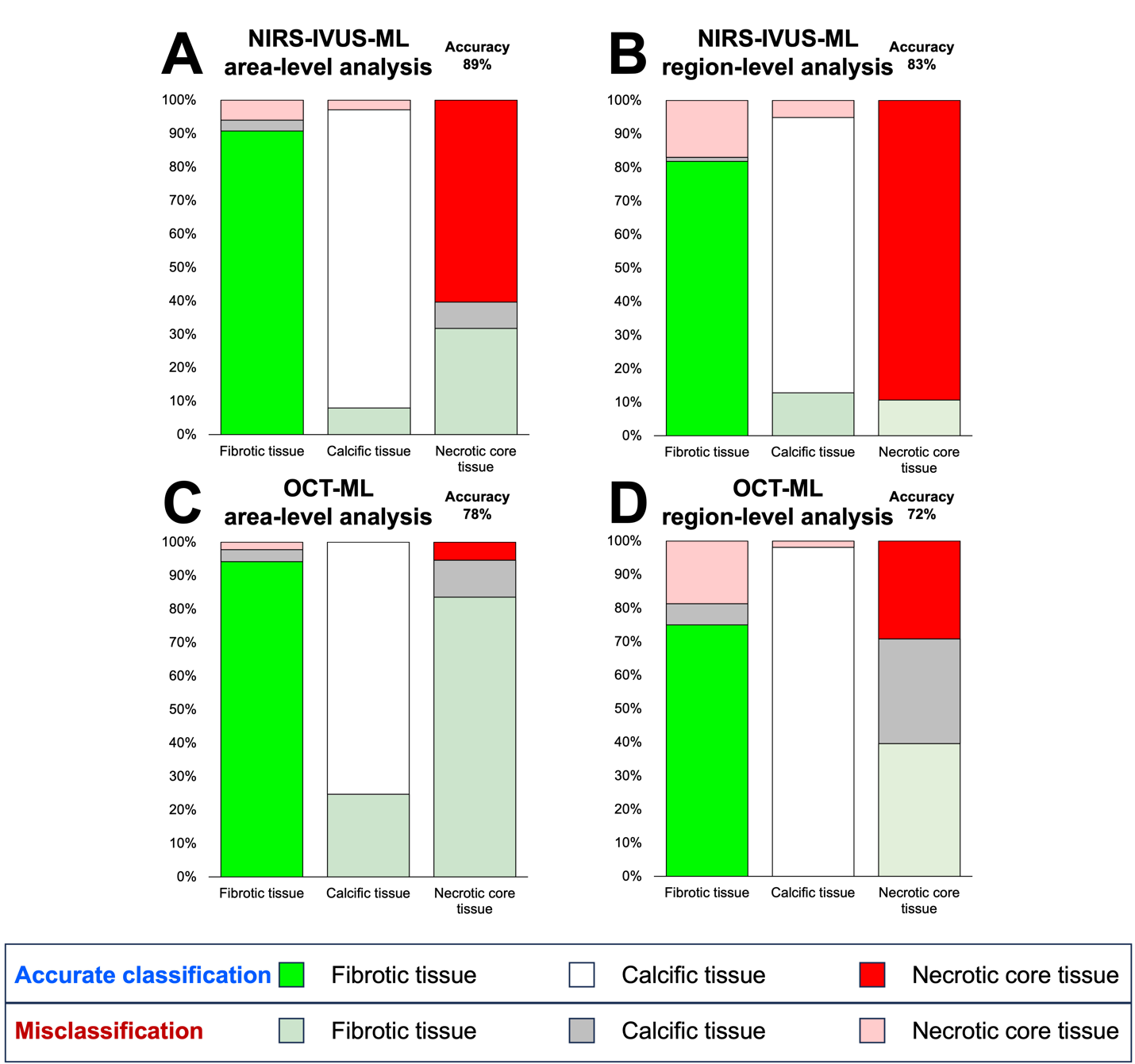


**Supplementary Figure 3.** Graphs comparing the plaque phenotype classification performance of NIRS-IVUS-ML and OCT-ML with histology for area-level (A & C) and region-level analysis (B & D).


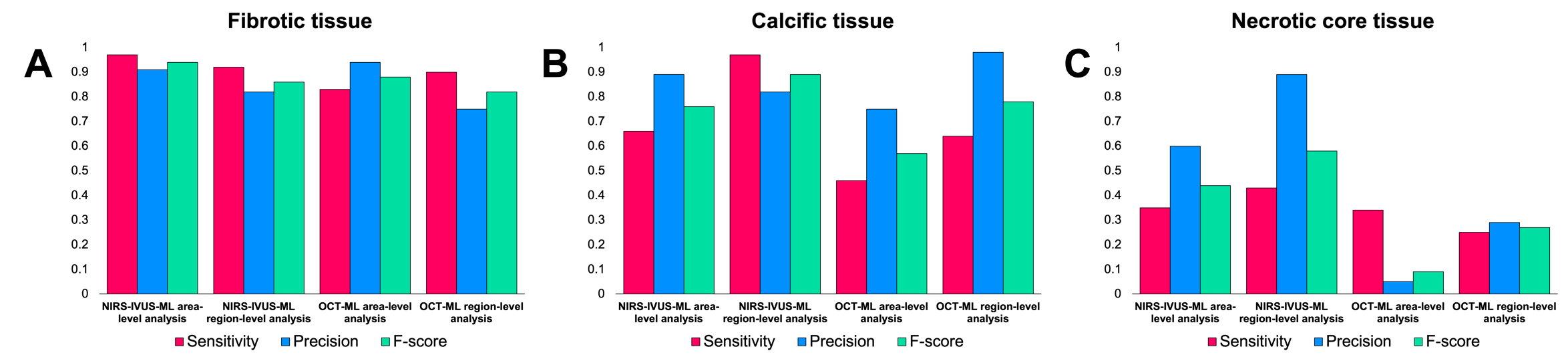


**Supplementary Figure 4.** Graphs comparing the area and region-level classification performance metrics of NIRS-IVUS-ML and OCT-ML for FT (A), Ca (B) and NC tissue (C).


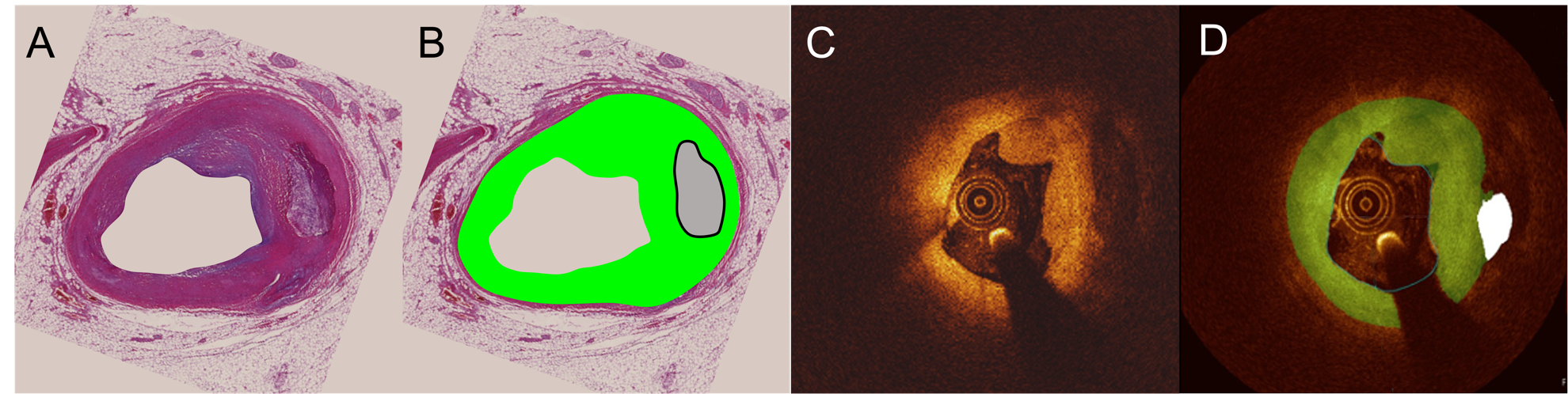


**Supplementary Figure 5.** Representative example illustrating incorrect interpolation of the EEM border by the OCT-ML classifier in a calcified plaque. The histology slide is shown in A, a mask showing fibrotic (green) and calcific (grey) plaque components is shown in B, the corresponding OCT in C and the output of the OCT-ML classifier in D (fibrotic plaque in green and calcific in white) where the deep calcific plaque and true EEM border has been misclassified.

**
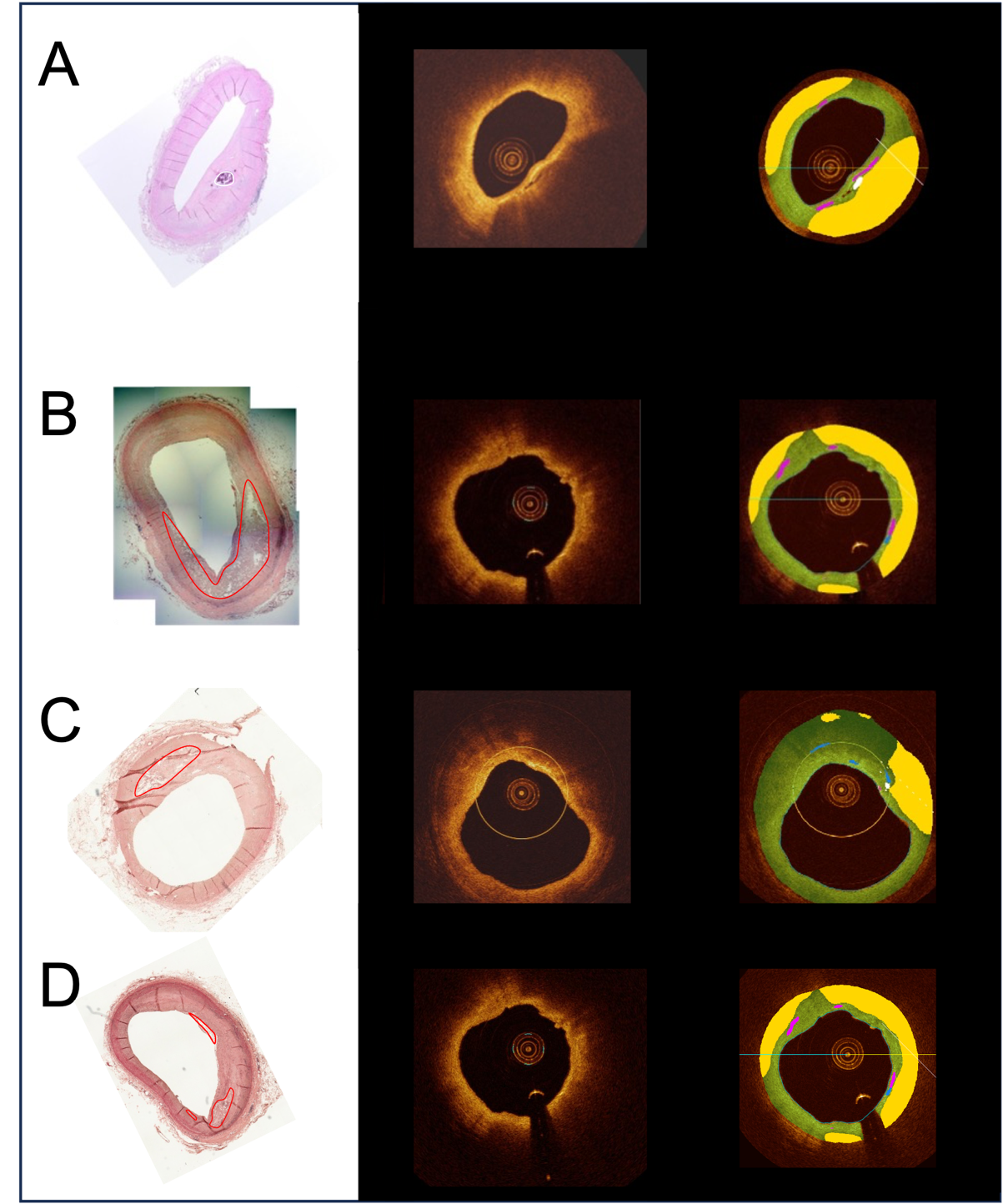
**

**Supplementary Figure 6.** Representative examples (A-D) illustrating typical classification errors of plaque components by the OCT-ML classifier. The histological slides are shown on the far left with annotation of plaque components (white as calcific tissue, red as necrotic core tissue), rotated to correspond to matched OCT frames shown in the middle of each panel. The output of the OCT-ML classifier on the right (fibrotic plaque in green, calcific in white, lipid in yellow, macrophages in purple).

**Supplementary Table 1.** Baseline demographics of the patients included in the NIRS-IVUS and OCT cohort.

|  | **NIRS-IVUS cohort**  **(n=12)** | **OCT cohort**  **(n=8)** | **P** |
| --- | --- | --- | --- |
| Age, years | 60±9 | 68±13 | 0.109 |
| Gender, male | 9 (75%) | 4 (50%) | 0.263 |
| **Medical history** |  |  |  |
| Diabetes | 7 (58%) | 0 (0%) | 0.009 |
| Dyslipidaemia | 3 (25%) | 1 (13%) | 0.505 |
| Smoking | 9 (75%) | 5 (63%) | 0.560 |
| Previous ACS | 5 (42%) | 1 (13%) | 0.174 |
| **Cause of death** |  |  |  |
| ACS | 0 (0%) | 0 (0%) | 1.000 |
| Other cardiac | 1 (8%) | 0 (0%) | 0.414 |
| Non-Cardiac | 11 (92%) | 3 (38%) | 0.012 |
| Unknown | 0 (0%) | 5 (63%) | 0.002 |

**Table footnote:** ACS, acute coronary syndrome; NIRS-IVUS, near-infrared spectroscopy intravascular ultrasound; OCT, optical coherence tomography.

**Supplementary Table 2.** Atheroma burden and plaque tissue component distribution in the histological datasets of the NIRS-IVUS and OCT cohorts.

|  | **NIRS-IVUS cohort**  **(n=131)** | **OCT cohort**  **(n=184)** | **P** |
| --- | --- | --- | --- |
| Average EEM area, mm^2^ | 13.43±4.89 | 11.53±5.01 | 0.001 |
| Average plaque area, mm^2^ | 7.03±3.06 | 7.00±2.66 | 0.926 |
| Average plaque burden, % | 51.92±12.20 | 63.89±11.71 | <0.001 |
| Histological sections with calcific tissue, n | 52 (39.7%) | 72 (39.1%) | 0.632 |
| Histological sections with necrotic core tissue, n | 44 (33.6%) | 75 (40.8%) | 0.195 |

**Table footnote:** EEM, external elastic membrane; NIRS-IVUS, near-infrared spectroscopy intravascular ultrasound; OCT, optical coherence tomography.

**Supplementary Table 3.** Overlapping and non-overlapping area analysis comparing the performance of NIRS-IVUS-ML and OCT-ML with histology for plaque area detection.

|  | NIRS-IVUS-ML  (n=131) | OCT-ML  (n=184) | P |
| --- | --- | --- | --- |
| Overlap of histology and ML-derived plaque area, % | 93.9±11.7 | 86.7±9.6 | 0.040 |
| Plaque area detected by ML that did not correspond to histology (false positive), % of histological plaque area | 12.6±9.9 | 3.7±3.2 | <0.001 |
| Histological plaque area not detected by ML (missed plaque area), % of histological plaque area | 4.8±4.7 | 14.5±9.1 | <0.001 |
| Total misclassification (false positive + missed plaque area), % of histological plaque area | 17.3±8.7 | 17.1±7.5 | 0.828 |

**Table footnote:** EEM, external elastic membrane; ML-machine learning; NIRS-IVUS, near-infrared spectroscopy intravascular ultrasound; OCT, optical coherence tomography.

**Supplementary Table 4.** Performance of OCT-ML in detecting macrophages in macrophage-rich regions in comparison with histology.

|  |  | **OCT-ML** | |
| --- | --- | --- | --- |
|  |  | **Macrophages (n=7)** | **No macrophages (n=60)** |
| **Histology** | **Macrophages (n=53)** | 7 | 46 |
|  | **No macrophages (n=14)** | 0 | 14 |

**Table footnote:** ML-machine learning; OCT, optical coherence tomography.
